# Evaluation of C-Reactive Protein Level in Patients with Pain Form of Temporomandibular Joint Dysfunction

**DOI:** 10.1155/2018/7958034

**Published:** 2018-04-08

**Authors:** Malgorzata Pihut, Piotr Ceranowicz, Andrzej Gala

**Affiliations:** ^1^Prosthodontics Department, Consulting Room of Temporomandibular Joint Dysfunction, Jagiellonian University Medical Collage, 4 Montelupich Str., 31-155 Krakow, Poland; ^2^Department of Physiology, Faculty of Clinical Physiology, Jagiellonian University Medical College, 16 Grzegorzecka Str., 31-531 Krakow, Poland

## Abstract

Temporomandibular joint dysfunction is a functional disorder concerned with the abnormal functioning of the muscles of the stomatognathic system and temporomandibular joints involved in the dynamic movements of the jaw and surrounding structures. The aim of the study was to compare the level of C-reactive protein in patients with pain and painless forms of temporomandibular joint dysfunction. *Materials and methods*. The study group consisted of 72 patients who reported to the prosthetic treatment because of temporomandibular joint dysfunction. The study group included 36 patients with pain form of dysfunction, and the control group included 36 patients with painless form of disorder. Each patient underwent specialized examination of functional disorders in order to diagnose the type of dysfunction and was commissioned to carry out a study of the blood test concerned with evaluation of the C-reactive protein (CRP) level in the same analytical laboratory. The results of the investigation were subjected to statistical analysis. The research obtained approval from the Ethics Committee of the Jagiellonian University (KBET/125/L/2013). Level of Evidence for primary research was established as type V. *Results*. The mean values of C-reactive protein levels in both groups were in the normal range and did not differ statistically significantly, which indicates the fact that the pain form of the temporomandibular joint disorders is not associated with inflammation of the soft tissues of the joint. *Conclusion*. Painful form of the temporomandibular joint dysfunctions is not connected with the inflammation of joints.

## 1. Introduction

Temporomandibular joint dysfunction includes dysfunction of the masticatory muscles of the stomatognathic system and temporomandibular joints and the surrounding structures. They are often associated with abnormal conditions of occlusion. Functional disorders do not include all disorders associated with musculoskeletal organs including inflammatory, degenerative arthritis, and cancer lesions of the muscles (multiple sclerosis, tetany, and dermatomyositis). They are often the result of muscles undergoing prolonged and excessive work and nonphysiological loads occurring in the stomatognathic system [1–4].

The pain form of the disease is manifested by spontaneous pain in the preaural region, accompanied by pain or tenderness of the masseter muscle. Pain that appears during palpation examination of temporomandibular joints is frequently not related to inflammation of the soft tissue around the temporomandibular joints, as it is claimed by several authors [1, 3, 5–9]. The cause of this problem is a long-term overload of the soft tissue causally associated with excessive muscle tension, that sometimes even persists for years [1–4, 6, 9].

C-reactive protein (CPR) was first isolated in the blood serum in 1930 by Tillet and Francis in a bacteriological laboratory in the Rockefeller Institute in New York. Long years of research on the protein allowed for its assessment in a clinical setting. CRP is synthesized primarily by hepatocytes and the Kupffer and Browicz cells and is a cyclic pentamer with a molecular mass of 120 kDa. Its expression was also found in monocytes and lymphocytes. Each of the subunits of the protein is composed of 206 amino acids noncovalently linked together [10–12]. Each amino acid has a single disulfide bridge formed by cysteines at positions 37 and 78. Among the modifying agents of CRP concentration are cytokines and interleukins 1 and 6. The mechanism of stimulation of the biosynthesis of CRP is still not fully understood. CRP is present in healthy individuals in an amount of 0–5 mg/l. There is no evidence of differences of this marker that depends on gender. Currently, it is recognized as an important marker of ongoing inflammation and cancer reaction in the body and one of the major proteins of the acute phase. Its concentration may be increased even thousand-fold within 48 hours of the onset of inflammation. CRP is used as one of the markers of choice in monitoring the acute phase response because the markers increase to a relatively high concentration compared to basal concentration [13–16].

Marking of C-reactive protein concentration is based on the immune reaction of monovalent antibodies against CRP using the methods of radial immunodiffusion, immunoelectrophoresis, immunonephelometry, radioimmunoassay, and enzyme immunoassay. In recent years, the methods are immunoblotting, ELISA assays, and techniques of modern molecular biology [12, 14].

The aim of the study was to evaluate the level of C-reactive protein in patients treated for the pain form of temporomandibular joint dysfunction.

## 2. Materials and Methods

The study enrolled a group of 72 patients, aged 21 to 48 years, of both sexes, who reported to the prosthodontic treatment in the Consulting Room of Temporomandibular Joint Dysfunction at the Dental Institute at the Jagiellonian University in Cracow, between January 2015 and February 2017, due to the pain and painless forms of the temporomandibular joint dysfunction, and acoustic symptoms, accompanied by excessive tension of the masticatory muscles. Symptoms persisted from 3 to 12 months prior to the beginning of the treatment. All patients were subjected to a specialist functional examination of the masticatory organ using typical procedures: information of spontaneous and provoked ailments, muscles and temporomandibular jaw palpation, functional manipulation, assessment of ranges of mandibular movements, joints' sounds, and dental rating. Patients were consecutively and alternately assigned to the study and control groups, 36 patients in each. The study group consisted of pain form of temporomandibular joint dysfunction and the control group included painless form of this dysfunction.

During the study, we analyzed information concerned with general health and present complaints, and a functional examination of the stomatognathic system was conducted, with particular regard to the nature and intensity of the perceived pain and discomfort of temporomandibular joints and masticatory muscles, which occurred prior to treatment and radiation of pain, the type of sound symptoms (clicks), volume, and the phase of the movement of lowering and lifting the jaw, during which there were tension-type headache and back pain (Table 1). Examination was conducted once.

Functional assessment of the masticatory apparatus and muscles consists of a medical interview and a physical examination. Another important element of a medical interview was to evaluate the patient's posture, facial expression, and the general well-being after the patient enters the surgery. Through a medical interview, detailed information about the current general health condition was revealed (including information about chronic and hereditary diseases); the examining physician obtained information about the patient's surgical treatment and head trauma history within the last 6–8 months and the current medications being taken on daily basis. In this context, particular attention was given to muscle and joint conditions, with a focus on multiple sclerosis, osteoporosis, fibromyalgia, trigeminal neuralgia, hormonal disorders, and autoimmune diseases.

Physical examination started with a facial symmetry test; the oral cavity was examined for the dental and periodontal status and the condition of oral mucosa. If the patient had any missing teeth, the number and location of existing natural teeth in the occlusion support zones were investigated. Dental classification (according to Eichner index or Galasińska–Landsberger classification) was recorded in the patient's medical file. Dental prosthetic restorations, if present, were examined in terms of their clinical value.

A clinical examination of temporomandibular joint consists of palpation and auscultation; palpation was performed simultaneously on both sides of the face by exerting a force of around 400 g per square centimeter. Auscultation was performed with the aid of a double-tube stethoscope. The individual palpation force was measured using an electronic kitchen scale [1, 3, 17, 18].

Factors of inclusion in the study were pain form of temporomandibular joint dysfunction, the required age range, and a good general state of health (particularly with regard to cardiac and metabolic disorders). Good general state of the patients' health means that patients did not suffer from the medical conditions that could have an influence on the level of CRP.

Factors for exclusion from the research included the will (consent) of the patient, the presence of general diseases, traumas, or local inflammations.

All respondents were directed to one external medical analysis laboratory to determine the level of C-reactive protein in the blood serum. For this purpose, the immunoturbidimetric assay was performed for the in vitro quantitative determination of CRP in human serum and plasma using Roche/Hitachi cobas c systems. It was also assumed that the correct level of this marker in patients who show no evidence of inflammation taking account of the above method is 0 to 5 mg/l [12, 14]. All patients underwent ultrasound examination of the temporomandibular joints.

In case of the measure giving the constant results, statistical analysis was based on the traditional methods of calculation: mean values, standard deviation, minimal values, maximal values, standard error of the mean, variance analysis for dependent variables, and post hoc Tukey test for dependent variables being the statistical significance measure.

To compare the dependencies between the results obtained in consecutive clinical tests, the nonparametric Friedman test, Kendall's W, and Wilcoxon signed-rank test (comparing two related samples) were used. For statistical studies, special computer software STATISTICA 2010 was used. A *p* value less than 0.05 was accepted as statistically significant. The research obtained approval from the Ethics Committee of the Jagiellonian University (KBET/125/L/2013). Clinical Trial Gov. Identifier was registered as well: NCT03065608.

Level of Evidence for primary research was established as type V.

## 3. Results and Discussion

The study involved 72 patients, ranging from 21 to 48 years, divided into two groups of 36 people. Group I included 24 women and 12 men (mean age 29), and group II control included 27 women and 8 men (mean age 31). Analysis of the results of clinical examinations conducted in both groups show a homogeneous clinical material (sound symptoms in the temporomandibular joint, range of motion of the jaw, abnormal range and symmetry of movement of the jaw, and difficulty in chewing foods) with the exception of pain, which is a factor differentiating patients between group I and II. The results of the clinical examination (symptoms indicating the occurrence of functional disorders) are summarized in Table 1.

**Figure 1:**
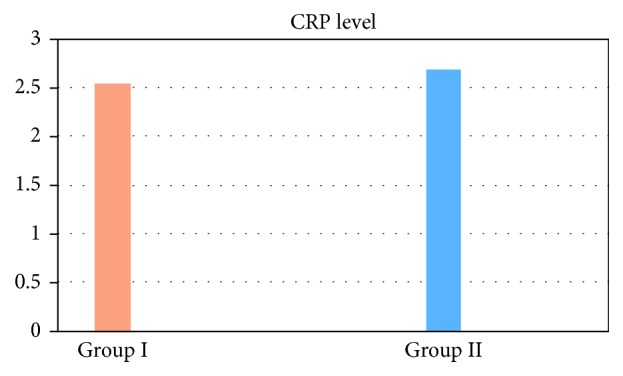
Average C-reactive protein values, obtained in both groups, presented graphically.

**Figure 2:**
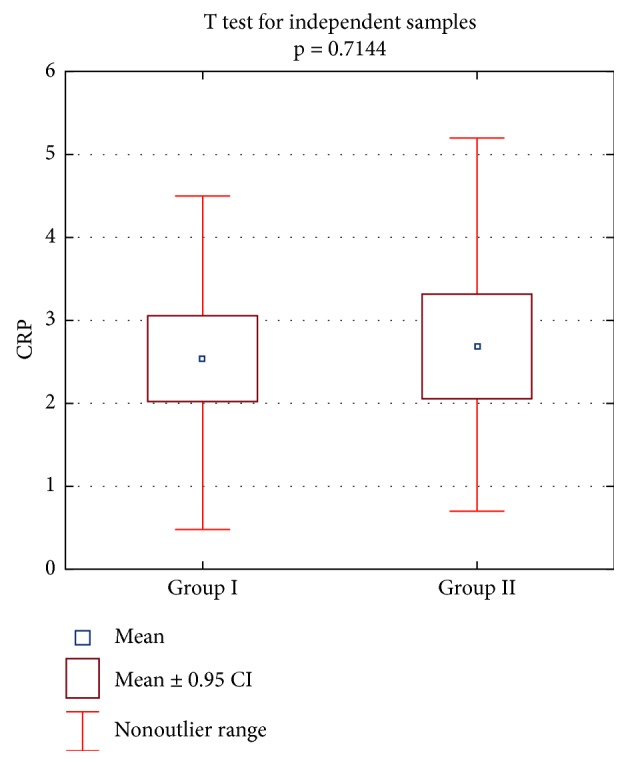
Average C-reactive protein values, obtained in both groups, presented in statistical evaluation.

In group I, the mean level of CRP was 2.54, the extreme values were 5.9–0.14, median was 2.15, and the standard deviation was 1.54. In group II, the average value of C-reactive protein was 2.69 (peak of 5.2–0.7), median was 2.4, and the standard deviation was 1.87. Average values of amounts of CRP did not significantly differ statistically, since *p* > 0.05. The results of the statistical calculations are graphically depicted in Figures 1 and 2.

None of the examined patients had signs of inflammation in the temporomandibular joints in ultrasound examination (pleural joint).

The authors of many publications [10–16, 19] emphasize the high utility of this marker in the diagnosis and confirmation of the presence of inflammation in the body, which also reflects a severity of current inflammation process. The level of this protein is increased between 6 and 8 hours after the pathogen in the body was found. The normalization of CRP is a valuable indicator of the patient's recovery. One should pay attention to general medical conditions concomitant to dysfunction because, for example, heart problems associated with the occurrence of continuous inflammation of the vascular endothelium will result in increase of CRP level and it will be due to the current associated medical main condition [19].

In the dental treatment specificity, the elevated level of CRP has been found in many diseases, such as periodontal disease, gangrenous pulp, fungal diseases of prosthetic base, or posttraumatic conditions—fractures of the jaws [12, 15]. While the states of inflammation of the temporomandibular joints are rare, and even more, they are not accompanied by temporomandibular joint dysfunction. They take the form of inflammation of the joint capsule (synovitis), the synovial membrane lining the inner surfaces of the bag, capsule ligaments (capsulitis), the retrodiscal tissues, and the area behind the articular disk (retrodiscitis) and inflammation associated with destructive changes in the bone (osteoarthritis). In each of these forms, the main causes of the inflammation for most parts are macrotrauma and mechanical damage. In contrast to functional disorders, when the pain associated with the movements of the mandible is dominant, inflammation is associated with continuous pain [1, 2, 20, 21].

CRP levels in our own study of both groups are in the normal range, and therefore this indicates that the pain form of temporomandibular joint dysfunction of subjects is not associated with the occurrence of the state of the inflammation in the soft tissues and bones of the temporomandibular joints. The presence of pain in the palpable test is often interpreted as an evidence of inflammation of tissues, but it is not confirmed in the clinical and laboratory tests.

Pain in any joint structure is called arthralgia. It would seem that pain is originated from the articular surfaces when the joints are overloaded by the work of muscles. It is impossible because of lack of the innervation of the articular surfaces, so arthralgia can originate only from nociceptors located in the soft tissue of the temporomandibular joints, like disc and capsular ligaments, and retrodiscal tissue [1–3, 16, 21, 22]. The temporomandibular articulation is unique in the body in that the two joints must always move simultaneously. Two distinct movements, rotation and translation, occur in the joint during mandibular opening and closing. Normal movements of the mandible depend on proper function of the temporomandibular joints. If the dysfunction is connected with muscles and joints prolonged overload, it will result as disruption of normal condyle-disc complex spatial relationship. In such a clinical form of dysfunction mainly the retrodiscal ligament is destroyed, and it is responsible for the occurrence of pain. The disc is then dislocated, mostly anteriorly. Meyer [23] describing the rules for the temporomandibular joint examination technique emphasizes that tenderness elicited by the palpation of the joint is invariably associated with joint inflammation.

In general, diseases such as arthritis and temporomandibular joint dysfunction can occur with elevated CRP because of general disease, as indicated by Lin et al. [24]. Nordahl et al. [16] described the progression of radiographic changes of the temporomandibular joint with reference to plasma levels of interleukin-1beta (IL-1beta), C-reactive protein (CRP), and disease duration. They point at the correlation of these factors with the severity of pathological changes. Progression of the overall grade of radiographic changes in the joints occurs in the group of the patients with chronic inflammatory joint disease. Increased levels of CRP are associated with progression of joints' bone loss.

Analyzing the available literature, it can be assumed that the use of studies evaluating CRP levels can be very helpful in diagnosing or excluding the inflammatory component of the temporomandibular joints. Kostrzewa-Janicka et al. indicate that more information could be obtained from synovial fluid of joints, like cytokines and their receptors, which are involved in the pathogenesis of temporomandibular joints. However, it is very difficult to aspirate the proper amount of synovial fluid from such a small joint, although recent advances in arthroscopic surgery allowed the direct examination of these joints [18]. These authors point out that in the case of joint diseases not related to functional disorders, the concentration of neuropeptides, cytokines, leukotrienes, prostaglandins, and catabolic products, identified in the joint's fluid, are correlated with joint pain and presence of surface lesions observed within the joint [18].

The above test results show the importance of information on the general principles of the treatment of pain form of temporomandibular joint dysfunction that the administration of antibiotics in case of pain on the palpation test of the temporomandibular joints may be wrong. Such a clinical situation requires careful verification and use of additional tests to plan an appropriate treatment [1, 2].

This provides valuable information for proper diagnosis and correct treatment in temporomandibular joint dysfunction with the use of occlusal splints and occlusal correction of the teeth contacts and supporting numerous treatments in physiotherapy, such as laser biostimulation, sonophoresis, physiotherapy, and manual therapy. Intraoral splints are commonly used in dental treatment for a variety of conditions. Such splints alter the condyle-disc-fossa relationship, probably by changing the loading status of joints and discs. The antibiotics are not necessary for such a kind of treatment [22].

Despite the etiological factor associated with the psychosocial sphere, this provides valuable information for proper diagnosis, and correct treatment of temporomandibular joint dysfunction is primarily occlusal splint, correction of occlusal contact, and numerous supporting physiotherapy treatments, such as biostimulation laser, sonophoresis, and manual techniques [1, 2, 5, 25–28].

## 4. Conclusion

Pain form of the temporomandibular joint dysfunctions is not connected with inflammation of the temporomandibular joints.

## Figures and Tables

**Table 1:** A summary of symptoms obtained during functional examination of stomatognathic system conducted in both groups.

Symptoms of temporomandibular joint disorders			Group I		Group II	
			W	M	W	M
Intrinsic pain	Single TMJ	Acute	14	6	0	0
		Dull	6	3	0	0
	Bilaterally	Acute	8	5	0	0
		Dull	1	2	0	0
Mastication pain or/pain presence during lower jaw movements			28	14	0	0
Pain of the TMJs triggered by palpation			20	13	0	0
Pain associated with lower jaw movements			28	17	0	0
Masticatory muscle pain			20	5	0	0
Radiation of pain			8	5	0	0
Sounds, snaps in TMJs	Single TMJ	R	9	1	8	3
		L	2	2	3	1
	Bilaterally	R	4	2	2	3
		L	4	2	2	3
Subjective feeling of increased tension in muscles			22	7	24	6
Constricted opening of the lower jaw			4	1	3	0
Constricted lateral mandibular movements			2	1	3	1
Mandibular deviation during opening			15	5	16	2
Difficulty in chewing			15	14	14	2
Occlusion parafunctions			20	8	21	5
“Closed ear” symptom on the affected TMJ side			2	2	4	2
Sudden hearing impairment			1	0	2	1
Tinnitus			2	0	1	1
